# Analysis of Social Communication Questionnaire (SCQ) Screening for Children Less Than Age 4

**DOI:** 10.1007/s40474-017-0122-1

**Published:** 2017-11-04

**Authors:** Alison R. Marvin, Daniel J. Marvin, Paul H. Lipkin, J. Kiely Law

**Affiliations:** 1 Department of Medical Informatics, Kennedy Krieger Institute, 3825 Greenspring Avenue, Baltimore, MD 21209 USA; 2 Wendy Klag Center for Autism and Developmental Disabilities, Johns Hopkins Bloomberg School of Public Health, 615 N. Wolfe Street, Baltimore, MD 21205 USA; 3 Department of Medical Informatics, Kennedy Krieger Institute, 733 N Broadway, Baltimore, MD 21205 USA

**Keywords:** Autism spectrum disorder (ASD), Social communication questionnaire (SCQ), Early childhood screening, Psychometric properties, Sensitivity and specificity, Area under ROC curve analysis

## Abstract

**Purpose of Review:**

The Social Communication Questionnaire (SCQ) is a screener for Autism spectrum disorder (ASD) validated for age 4.0 +. There is a clinical need for an ASD screener for children beyond the 30-month age limit of the M-CHAT-R/F. We evaluate the literature on the use of the SCQ in children < 4.0 years.

**Recent Findings:**

Recent studies have used very large samples; included typically developing children, rather than just those with developmental disorders; compared the SCQ Lifetime and Current versions; and increased scrutiny of internal validity.

**Summary:**

The sensitivity-specificity balance in distinguishing between ASD and other developmental disorders is poor, which has led to development of abbreviated versions of the SCQ; however, sensitivity-specificity balance is better in a more general population. The SCQ Lifetime (not Current) version should be used. Future research relating should focus on further validation of the SCQ as a screener for children 30–48 months.

## Introduction

Autism spectrum disorder (ASD) currently affects approximately 1 in 68 children across the USA, and is about 4.5 times more common among boys than among girls [1].

Children can be reliably diagnosed with ASD by age 2 [2–7], clearing a pathway to early intervention opportunities. Early intervention may not only limit deterioration of skills, but may lead to such improvement in functioning, such that children with higher intelligence and functional skills may later lose their ASD diagnosis [8–10].

The American Academy of Pediatrics (AAP) recommends screening for ASD at both 18- and 24-month well visits [11–13] with an autism-specific screening tool, such as the Modified Checklist for Autism in Toddlers, revised with follow-up (M-CHAT-R/F) [14]. The AAP also recommends screening for ASD at later visits in cases where there is concern; however, the M-CHAT-R/F is only valid for ASD screening of toddlers between 16 and 30 months of age.

There is, thus, a clinical need to have an ASD screening instrument with acceptable psychometric properties for children between 30 and 48 months. It must be stressed that a screening tool cannot be used to make a diagnosis; if the child fails on the screening instrument, he or she is referred to a specialist to receive a comprehensive clinical evaluation to determine a diagnosis, if any. If the screening instrument fails too many children, the referral system may become overwhelmed with concomitant delays for children in receiving a diagnosis; however, if the screening instrument does not screen positive for the child who truly does have ASD, the child will not be sent for evaluation to receive a diagnosis of ASD and so will not receive intervention services.

The Social Communication Questionnaire (SCQ) [15, 16] offers a screening instrument for ASD that has been validated for children age 4.0 years or older. The goal of this paper is to critically evaluate the literature on the use of the Social Communication Questionnaire (SCQ) for children under age 4.0 years, with special reference to recent findings.

## Social Communication Questionnaire (SCQ): a Screening Measure for ASD

The SCQ is a brief, 40-item, parent-report screening measure that focuses on items relating to ASD symptomatology likely to be observed by a primary caregiver. Although the SCQ is a screening tool—and, thus, cannot be used for diagnosis of ASD—it is based on the Autism Diagnostic Interview (ADI-R) [17], a semi-structured parent interview conducted by a trained clinician or researcher that can be used for diagnostic evaluation of children with suspected ASD.

Each item in the SCQ requires a dichotomous “yes”/“no” response, and each scored item receives a value of 1 point for abnormal behavior and 0 points for absence of abnormal behavior/normal behavior. The first item—“Is she/he now able to talk using short phrases or sentences?”—is not scored, but rather determines whether six items relating to abnormal language are assigned. Only “verbal” children (i.e., children with a “yes” response to the first question) are assigned the six items relating to abnormal language and can, thus, can score a total of 0–39 points; “non-verbal” children (i.e., children with a “no” response to the first question) are not assigned the six items in relation to abnormal language and so can score a total of 0–33 points.

There are two different versions of the SCQ. The SCQ Current asks respondents to indicate whether behaviors have been present during the past 3 months. By contrast, the SCQ Lifetime references complete developmental history and asks respondents to indicate whether behaviors have ever been present for questions 2–19 and whether behaviors were present at age 4 years—or to consider behavior in the past 12 months if the child is not yet 4 years—for questions 20–40.

Agreement between the SCQ and the ADI-R at the total score level is high [17]. Per its authors, the SCQ is applicable to subjects of “any chronological age above age 4.0 years provided that their mental age is at least 2.0 years” [17]. The authors suggest that the applicability of the ADI-R may extend down to a chronological age of 2.0 years as long as the mental age exceeds 2.0 years, but caution against using the SCQ in subjects under age 4.0 years because data for that population had not yet been systematically tested and no individuals under age 4.0 were included in the sample used in the development of the SCQ. Several studies have indicated that the SCQ is not age neutral and that the measure performs better in older children [18, 19•], which may be problematic in attempting to use the SCQ in children under 4.0 years.

## Literature Selection

Studies incorporated into this review were drawn from keyword-guided online searches on PubMed, Google Scholar, and the International Meeting for Autism Research online archives. Studies must specifically report on the psychometric properties of the SCQ Lifetime and/or SCQ Current for children under age 4.0 years. In addition, studies must been published 2003 or later, corresponding to the year that the SCQ was released.

Eight studies were selected for inclusion [20–25, 26••, 27••]. One study [23] included 97% of the sample from another study [22] by the same authors. In addition, high-level unpublished summary data from the current authors (in manuscript) have been included [28••].

Six early studies (2007–2010) are listed in Table 1 [20–25] and three current studies (2016–2017) are listed in Table 2 [26••, 25••, 28••]. The tables include the following: the authors and years of publication in alphabetical order; information on how each sample was selected; the age range of the sample population; sample characteristics; and which version of the SCQ was used. Tables also include key psychometric data, which are important for understanding the value of the SCQ in this population. Psychometric data are described in the next section.

**Table 1:** External validity studies of the Social Communication Questionnaire (SCQ) in children under age 4 using receiver operating characteristic (ROC) curve analysis: early studies (2007–2010)

Author (year)	Sample selection	Age range (months)	Sample characteristics (*n*)	Cutoff	Sensitivity	Specificity	AUC	LR+	LR−	Youden’s J	SCQ version
Allen et al. (2007) [20]	Clinical sample referred for developmental/behavioral assessment	24–36	Diagnosis not specified (16)	15	0.56	0.29	–	0.79	1.52	−0.15	Current*
				11	0.89	0.29		1.25	0.38	0.18	
		37–48	Diagnosis not specified (30)	15	0.82	0.79	–	3.90	0.23	0.61	
				11	1.00	0.58		2.38	0.00	0.58	
Lee et al. (2007) [21]	Children receiving preschool public spec. ed. services	36–47	ASD (10) vs. non-ASD developmental delay (68)	15	0.70	0.88	0.88	5.93	0.34	0.58	Current
				11	0.60	0.94		10.17	0.43	0.54	
				14	0.80	0.85		5.44	0.23	0.65	
Oosterling et al. (2009) [22]	Screened high-risk clinical sample referred for assessment	8–24	ASD (35) vs. non-ASD: psychiatric disorder (9)/no problem (2)	11	0.89	0.27	0.71	1.22	0.41	0.16	Current
				15	0.74	0.55	0.71	1.64	0.47	0.29	
		25–44	ASD (125) vs. non-ASD psychiatric disorder (67)	11	0.83	0.28	0.66	1.15	0.61	0.11	
				15	0.63	0.66		1.85	0.56	0.29	
Oosterling et al. (2010) [23]	Toddlers referred for clinical assessment	20–40	ASD (151) vs. non-ASD (66)	20	0.39	0.80	0.64	1.95	0.76	0.19	Current
				18	0.50	0.70		1.67	0.71	0.20	
				14	0.70	0.54		1.52	0.56	0.24	
				11	0.80	0.25		1.07	0.80	0.05	
Snow and Lecavalier (2008) [24]	Clinical sample referred for possible ASD	30–48	ASD (29) vs. non-ASD developmental delay and/or language impairment (10)	15	0.72	0.30	0.62	1.03	0.92	0.02	Current
Wiggins et al. (2007) [25]	Clinical sample referred to early intervention program	17–45	ASD (19) vs. non-ASD developmental delay (18)	15	0.47	0.89	–	4.27	0.60	0.36	Current*
				13	0.68	0.89		6.18	0.36	0.57	
				11	0.89	0.89		8.09	0.12	0.78	

Cutoff. For each SCQ cutoff value, a positive or negative ASD diagnosis is made for each child by comparing the SCQ Total Score to the select SCQ cutoff value

Sensitivity (true positive rate) and specificity (true negative rate). Values: 1.0 = perfect; 0.9–1.0 = very good; 0.8–0.9 = good; 0.7–.0.8 = fair; <0.7 = poor

AUC. Area under the receiver operating characteristic (ROC) curve; plots sensitivity against 1-specificity. A measure of how well the SCQ Total Score can distinguish between the presence of an ASD diagnosis and the absence of an ASD diagnosis. Values: 1.0 = perfect; 0.9–1.0 = very good; 0.8–0.9 = good; 0.7–.0.8 = fair; 0.6–0.7 = poor; 0.5–0.6 = very poor; 0.5 = non-discriminating. Values below 0.5 mirror those with values above, with 0.0 = perfect inverse

LR+. Likelihood ratio for positive test results; an indicator for ruling-in ASD. Calculated as sensitivity/(1-specificity). Higher is better; good diagnostic tests have LR+ > 10

LR−. Likelihood ratio for negative test results; an indicator for ruling out ASD. Calculated as (1-sensitivity)/specificity. Lower is better; good diagnostic tests have LR− < 0.1

Youden’s J. An indicator that gives equal weight to sensitivity and specificity, and which is often used to establish the optimal cutoff point. It is calculated as Sensitivity + Specificity − 1. Values: 1 = perfect; 0 = no value; − 1 = perfect inverse

* Per personal correspondence with lead researcher

**Table 2:** External validity studies of the Social Communication Questionnaire (SCQ) in children under age 4 using receiver operating characteristic (ROC) curve analysis: current studies (2016–2017)

Author (year)	Sample selection	Age range (months)	Sample characteristics (*n*)	Cutoff	Sensitivity	Specificity	AUC	LR+	LR−	Youden’s J	SCQ version
Barnard-Brak et al. (2016) [26••]	Secondary data from NDAR for children with confirmatory ADOS scores	< 48	Not specified	*Analysis not performed on full SCQ*							Lifetime where available, else Current
Day et al. (2017) [27••]	Positive and negative screens in primary care	24–47	ASD (181) vs. 203 non-ASD (203) [DD = 91 vs. TD = 112]	15	0.41	0.86	0.69	2.86	0.69	0.27	Lifetime
				7	0.81	0.43		1.42	0.44	0.24	
Marvin et al. (unpublished) [28••]	Children with ASD and siblings without ASD in a verified parent-report online ASD research registry	24–47	ASD verbal (978) vs. non-ASD verbal siblings (1320)	15	0.85	0.96	0.97	19.36	0.15	0.81	Lifetime
				12	0.93	0.93		13.41	0.08	0.86	
				10	0.96	0.90		9.22	0.05	0.86	
			ASD non-verbal (1195) vs. non-ASD non-verbal Siblings (185)	15	0.91	0.81	0.90	4.64	0.12	0.71	
				12	0.97	0.70		3.25	0.05	0.67	
				11	0.98	0.67		2.92	0.03	0.64	

Cutoff. For each SCQ cutoff value, a positive or negative ASD diagnosis is made for each child by comparing the SCQ Total Score to the select SCQ cutoff value

Sensitivity (true positive rate) and specificity (true negative rate). Values: 1.0 = perfect; 0.9–1.0 = very good; 0.8–0.9 = good; 0.7–.0.8 = fair; < 0.7 = poor

AUC. Area under the receiver operating characteristic (ROC) curve; plots sensitivity against 1-specificity. A measure of how well the SCQ Total Score can distinguish between the presence of an ASD diagnosis and the absence of an ASD diagnosis. Values: 1.0 = perfect; 0.9–1.0 = very good; 0.8–0.9 = good; 0.7–.0.8 = fair; 0.6–0.7 = poor; 0.5–0.6 = very poor; 0.5 = non-discriminating. Values below 0.5 mirror those with values above, with 0.0 = perfect inverse

LR+. Likelihood ratio for positive test results; an indicator for ruling-in ASD. Calculated as sensitivity/(1-specificity). Higher is better; good diagnostic tests have LR+ > 10

LR−. Likelihood ratio for negative test results; an indicator for ruling out ASD. Calculated as (1-sensitivity)/specificity. Lower is better; good diagnostic tests have LR− < 0.1

Youden’s J. An indicator that gives equal weight to sensitivity and specificity, and which is often used to establish the optimal cutoff point. It is calculated as Sensitivity + Specificity − 1. Values: 1 = perfect; 0 = no value; − 1 = perfect inverse

## Psychometric Data Relating to Receiver Operating Characteristic Curve Analysis

The following psychometric data, which are included in Tables 1 and 2, represent key results of receiver operating characteristic (ROC) curve analysis, and are described below.

ROC curve analysis—including key measures of sensitivity, specificity, and area under the ROC curve—is associated with test validity. Generally, validity refers whether the test measures what it was intending to test and provide information about the accuracy of the test. The SCQ would be described as having criterion validity if its results are associated with the “gold standard” of, say, a clinical assessment.

*Cutoff*: for each SCQ cutoff value, a positive or negative ASD diagnosis is made for each child by comparing the SCQ Total Score to the select SCQ cutoff value.

*Sensitivity (true positive rate)*: the proportion of children with ASD who are correctly identified as having ASD, which also quantifies the avoidance of false negatives (type II error: children with ASD being incorrectly identifying as not having ASD). Values: 1.0 = perfect; 0.9–1.0 = very good; 0.8–0.9 = good; 0.7–.0.8 = fair; < 0.7 = poor.

*Specificity (true negative rate)*: the proportion of children without ASD who are correctly identified as not having ASD, which also quantifies the avoidance of false positives (type I error: children without ASD being incorrectly identifying as having ASD). Values: 1.0 = perfect; 0.9–1.0 = very good; 0.8–0.9 = good; 0.7–.0.8 = fair; < 0.7 = poor.

Different cutoff scores express the trade-off between sensitivity and specificity, which can be represented graphically by a receiver operating characteristic (ROC) curve, which plots sensitivity (the true positive rate) on the *y*-axis against 1-specificity (the false positive rate) on the *x*-axis. A key goal of ROC curve analysis is the select an optimal cutoff; however, there are likely to be different optimal cutoffs dependent on the purpose of the researcher or clinician.

*Area under the ROC curve*: a measure of how well the SCQ Total Score can distinguish between the presence of an ASD diagnosis and the absence of an ASD diagnosis. AUC varies between 0 and 1 (in normalized units), where AUC = 1.0 represents a perfect diagnostic accuracy; AUC between 0.9 and 1.0 represents very good diagnostic accuracy; AUC between 0.8 and 0.9 represents good diagnostic accuracy; AUC between 0.7 and 0.8 represents fair diagnostic accuracy; AUC between 0.6 and 0.7 represents poor diagnostic accuracy; AUC between 0.5 and 0.6 represents very poor accuracy; and AUC = 0.5 represents a non-discriminating test [29].

Likelihood ratios (LR), below, have been calculated by the current authors from researcher-reported sensitivity and specificity data. LR provide a statistic about test reliability, that is the degree to which an assessment tool produces stable and consistent results, that is independent of ASD prevalence in the population tested. (By contrast, positive predictive value (PPV) and negative predictive value (NPV) are measures of reliability that, indeed, are affected by disease prevalence.)

*Likelihood ratio for positive test results (LR+)*: an indicator for ruling-in diagnosis of ASD where higher is better, which is calculated as sensitivity/(1-specificity). Good diagnostic tests have LR+ > 10.

*Likelihood ratio for negative test results (LR−)*: an indicator for ruling out ASD where lower is better, which is calculated as (1-sensitivity)/specificity. Good diagnostic tests have LR- < 0.1.

The authors also calculated Youden’s J statistic, below, from researcher-reported sensitivity and specificity data. Youden’s J which is a single statistic that summarizes the performance of a dichotomous test and represents “informedness,” the probability of an informed decision or, as Powers [30] conceptualizes it, the “edge” a punter has in making his bet as quantified by his winnings.

*Youden’s J*: an indicator that which is often used to establish the optimal cutoff point. It gives equal weight to sensitivity and specificity. It is calculated as Sensitivity + Specificity − 1, and has values from + 1 (indicating a perfect measure in which there are no false positives or negatives) to − 1 (indicating a perfect inverse measure). A value of 0 indicates that the measure has no value [31].

## Comparing Early (2007–2010) and Current (2016–2017) Studies

Children included in the six early studies (see Table 1) received a full ASD assessment in conjunction with administration of the SCQ Current. Five studies [20, 22–25] included children who had been referred for assessment, while one [21] included children receiving preschool special education services; thus, children are generally categorized into ASD diagnosis vs. non-ASD psychiatric and/or developmental and/or behavioral diagnoses. Samples sizes for these early studies were small, and focused on the use of receiver operating characteristic (ROC) analyses to determine the ability of the SCQ to distinguish between the presence or absence of ASD at the recommended SCQ threshold of 15 and at other cutoff points.

The three current studies (2016–2017) which are the main focus of this paper—Barnard-Brak et al. [26••], Day et al. [27••], and Marvin et al. [28••]—are listed in Table 2.

Result for ROCs analyses of the full SCQ for both early and current studies can be found in Tables 1 and 2, respectively. All but one study [26••] performed ROC curve analysis on the full SCQ measure.

All three current studies:

*Used the SCQ Lifetime*: one study [26••] used both the SCQ Lifetime and the SCQ Current, but gave precedence to the SCQ Lifetime where available. The authors of the SCQ designed the SCQ Current to focus on changes over time in individuals previously diagnosed with ASD [16]; however, they also suggested using the SCQ Current as an alternative to the SCQ Lifetime among young children [21]. Wei et al. [32••] compared the psychometric properties of the SCQ Lifetime and the SCQ Current, and strongly caution against the general use of the SCQ Current due to numerous measurement issues, and specifically state that “it seems inappropriate to use the Current form as an alternative to the Lifetime form among children younger than 5 years old” due to its problematic nature.

*Include typically developing children*: rather than exclusively children with psychiatric and/or developmental and/or behavioral concerns; thus, exploring the potential for routine clinical screening. Day et al. included both positive and negative screens in a primary care setting, while Marvin et al. included unaffected siblings of children with ASD. This broadens the external validity of the SCQ and allows generalizability to populations beyond those with developmental concerns.

*Have large sample sizes*: Day et al. included 384 children ascertained through screening in primary care and is the largest study to date for this age group in which each child received a full ASD assessment in conjunction with administration of the SCQ. Both Barnard-Brak et al. and Marvin et al. used data from large research databases, with Marvin et al. being the largest study to date of the use of the SCQ in young children, with 3678 fully completed SCQ forms—large enough to split analyses by both verbal/non-verbal status (based on the first question of the SCQ) and by age in years (2 vs. 3 years).

*Extensively reviewed internal consistency and validity of the SCQ Lifetime*: Day et al. and Marvin et al. also analyzed external validity using ROC analysis for the full SCQ Lifetime.

- Barnard-Brak et al. used Item Response Theory (IRT) Mokken scaling analyses to create an abbreviated scale, and performed confirmatory factor analysis and ROC curve analyses on the abbreviated scale. They found that bootstrapped ROC curve analyses of a 7-item version of the SCQ provide an AUC value of .81. Sensitivity was 0.67 and specificity was 0.75 at the cutoff of 3, with a Youden’s J value of 0.52. The 7 items were the following: socially inappropriate questions/statements (Q4); used other’s hand like a tool (Q10); odd, preoccupying interests (Q11); unusual, intense special interests (Q13); odd ways or movements (Q15); looking directly at you in communicating (Q26); and reciprocal imaginative play (Q39).
- Day et al. used ROC analysis to develop abbreviated SCQ measures and found that ROC curve analysis of 6 items had an AUC > .757, with sensitivity of 88.9% and specificity of 39.1% at a cutoff of 1. The 6 items were the following: reciprocal conversations (Q2); unusual hand and body mannerisms (15–16); nodding head for “yes” (24); offering comfort (31); and reciprocal imaginative play (Q39).
- Marvin et al. found that the full SCQ had high internal consistency (with Cronbach’s alpha of .94 for verbal children and .89 for nonverbal children). Marvin et al. also performed confirmatory factory analysis on the original four factors of the SCQ (Factor 1: Social Interaction; Factor 2: Communication; Factor 3: Abnormal Language; Factor 4: Stereotyped Behavior), but found that Factor 1: Social Interaction did not hold in this population.

The two studies [26••, 28••] that used secondary data analysis without incorporating full assessments into their respective studies used alternate methods of confirming ASD diagnosis in their population.

Barnard-Brak et al. used the National Database for Autism Research (NDAR) [33], an NIH-funded research data repository, and selecting a sample where there was a confirmatory Autism Diagnostic Observation Schedule (ADOS) [34].

Marvin et al. used participant-report data from the Interactive Autism Network (IAN) [35], an online autism research registry and database. The IAN registry includes the proband child with ASD, who is required to have a professional diagnosis of ASD, and his or her parents and siblings. IAN uses the SCQ Lifetime for confirmation of parent-report of professional diagnosis of ASD, rather than as a screening instrument. Parental report of professional diagnosis of ASD has been verified by medical records [36]. Community professional diagnosis of ASD has been clinically validated for both verbal children aged 4–17 years [37] and non-verbal children aged 6–17 [38] with SCQ Lifetime scores ≥ 12, where dichotomous verbal/non-verbal status was determined based on the response to the first question of the SCQ Lifetime. Marvin et al. analyzed a subset of data for children who had a re-confirmed their diagnosis at age 6 years or older via completion of updated baseline development questionnaire in order to account for children with ASD might have been incorrectly diagnosed at such a young age and unaffected siblings who might not yet have been diagnosed. The results of the analyses on the subset data were improved, but generally comparable to the score on the full dataset.

Studies generally recommended a cutoff below the threshold of 15 for children under age 4.0. Most studies advised selecting a cutoff to emphasize sensitivity or specificity depending on need because the balance between sensitivity and specificity was generally poor. For example, a cutoff score that provided high sensitivity would generally have low specificity, resulting in a large false positive rate.

Rather than use the SCQ as a screener, Marvin et al. used the SCQ for confirmation of parent-report of diagnosis. The researchers differentiated between children with ASD and their generally typically developing siblings (although siblings are at higher risk of ASD than the general population), rather than children with non-ASD concerns. They obtained very good results for verbal children (sensitivity = .93; specificity = .93; AUC = .97; LR + =13.41; LR − = .05) with a cutoff of 12, and good results for non-verbal children (sensitivity = .91; specificity = .81; AUC = .90; LR + =4.64; LR − = .12). The lower performance for the non-verbal children is likely due to the association between non-verbal status and intellectual disability and, thus, the mental age of the non-verbal children might not be at the 2.0-year level. See Table 2 for additional results at different cutoff points.

## Concluding Remarks

Recent studies of the use of the SCQ in younger children have focused on the following: the use of large samples, including secondary data analysis from large research datasets; the inclusion of typically developing children in analyses, rather than limiting analyses to children with ASD and non-ASD concerns; comparative psychometric analysis of the SCQ Lifetime and Current versions; and the increased scrutiny of internal validity, which has led to development of abbreviated versions of the SCQ.

Those interested in using the SCQ in children younger than the recommended age of 4.0 years should use the Lifetime version, rather than the SCQ Current, due to the poor psychometric properties of the SCQ Current in this age group.

The sensitivity-specificity balance is poor in a population where children have a diagnosis, whether ASD or non-ASD; thus, development of an abbreviated version may be useful for high-risk children for differentiating between those with ASD and those with another developmental diagnosis. The sensitivity-specificity balance was better in a general population comparing children with ASD to (generally) typically developing children. In addition to showing potential as a screening tool for ASD in children younger than 4.0 years in the general population, the SCQ Lifetime has also been demonstrated to confirm diagnosis of ASD in young children who have already received a diagnosis of ASD. The cutoff score should be adjusted per the user’s goals to prioritize sensitivity or specificity.

Screening for ASD in young children is complex. Recent studies [39–42] have suggested that it might not be possible to appropriately screen children as young as 18 months, or even 24 months. Children with overt developmental issues, including delayed speech and lower functioning, are more likely to be sent for evaluation by 24 months. Given the strong potential of using the SCQ Lifetime for screening higher-functioning children below age 4.0 years, future research relating to the SCQ should also focus on further validation of its use as a screener for children between 30 and 48 months.
